# Bone cement implantation syndrome: a scoping review

**DOI:** 10.1016/j.bja.2025.05.041

**Published:** 2025-07-08

**Authors:** Karen E. Brokke, Manon Graman, Sjoerd Servaas, Inger N. Sierevelt, Monique A.H. Steegers, Peter A. Nolte

**Affiliations:** 1 Department of Orthopaedic Surgery, Spaarne Gasthuis Academy, Hoofddorp, The Netherlands; 2 Department of Anaesthesiology, Amsterdam Movement Sciences, Amsterdam University Medical Centers, Amsterdam, The Netherlands; 3 Department of Anaesthesiology, University Medical Centers Utrecht, Utrecht, The Netherlands; 4 Department of Anaesthesiology, Spaarne Gasthuis Academy, Hoofddorp, The Netherlands; 5 Department of Orthopaedic Surgery and Sports Medicine, Amsterdam Movement Sciences, Amsterdam University Medical Centers, Amsterdam, The Netherlands; 6 Department of Orthopaedic Surgery, Xpert Clinics, Amsterdam, The Netherlands; 7 Department of Oral Cell Biology, Academic Centre for Dentistry, University of Amsterdam and Vrije Universiteit Amsterdam, Amsterdam, The Netherlands

**Keywords:** bone cement implantation syndrome, cementation, cemented arthroplasty, desaturation, hypotension, hypoxia, uncemented arthroplasty

## Abstract

**Background:**

Bone cement implantation syndrome comprises the occurrence of hypoxaemia, hypotension, unexpected loss of consciousness, or cardiac arrest occurring around the time of cementation, prosthesis insertion, or reduction of the joint in arthroplasty. As the yearly number of arthroplasties will increase, the number of complications including bone cement implantation syndrome is also expected to increase. Therefore, a good understanding of this syndrome is important. This scoping review aims to provide a comprehensive overview of the current knowledge of bone cement implantation syndrome.

**Methods:**

This scoping review was conducted based on the PRISMA-ScR Checklist. A literature search was done in PubMed, Cochrane, and Embase. A total of 85 studies were included in the study.

**Results:**

The incidence of bone cement implantation syndrome during cemented hip or knee arthroplasty varied from 15.4% to 46.7% and 27.5% to 70.7%, respectively. The incidence in shoulder arthroplasty was 16.2%. In uncemented hip arthroplasty, the incidence ranged from 0.0% to 21.8%. Risk factors identified for bone cement implantation syndrome included advanced age, ASA physical status 3 or 4, and primary lung cancer or lung metastasis.

**Conclusions:**

Bone cement implantation syndrome is a potentially severe complication that can occur during both cemented and uncemented arthroplasty. Moreover, the occurrence of bone cement implantation syndrome is not dependent on the use of cement. However, the severity does seem to have an association with the fixation method.

**Editor’s key points**

• Bone cement implantation syndrome is characterised by hypoxaemia and hypotension. Largely, it has been assumed to be exclusive to cemented hip and knee arthroplasty.
• This review shows that bone cement implantation syndrome also occurs in uncemented procedures, although it appears to be less severe than in cemented arthroplasty. Risk factors include advanced age, ASA physical status 3-4, and pulmonary malignancy.
• Further research is needed on uncemented arthroplasty, and on prevention and treatment strategies. A more detailed definition is proposed.

Cardiac arrest around the time of cementation during arthroplasty is a feared complication that surgery team members encounter perhaps once during their career. It is one of the symptoms of the syndrome known as the bone cement implantation syndrome (BCIS). This syndrome is characterised by hypoxia, hypotension, unexpected loss of consciousness, or cardiac arrest occurring around the time of cementation, prosthesis insertion, or reduction of the joint. Donaldson and colleagues^1^ classified the syndrome according to the severity of symptoms: grade 1, moderate hypoxaemia (SpO_2_ <94%) or hypotension (decrease in systolic blood pressure [SBP] >20% and <40%); grade 2, severe hypoxaemia (SpO_2_ <88%) or hypotension (decrease in SBP >40%) or unexpected loss of consciousness; and grade 3, cardiovascular collapse requiring cardiopulmonary resuscitation.

Initially, BCIS was believed to be exclusive to cemented hip and knee arthroplasty. However, emerging evidence suggests that uncemented arthroplasty may also result in similar clinical manifestations.^2^ Furthermore, BCIS is not limited to hip or knee arthroplasty but has also been reported in other procedures, such as shoulder arthroplasty.^2^ It is expected that in the coming years, the number of hip, knee, and shoulder arthroplasties will increase, driven in part by an ageing population and increasing obesity rates.^3^, ^4^, ^5^, ^6^, ^7^ Consequently, the number of patients with BCIS is also projected to increase. Given the potential severity of this syndrome, it is important to enable prompt recognition and appropriate management. Therefore, clinicians need to be well-informed about its pathophysiology, risk factors, and clinical presentation. In this scoping review, we investigate the incidence of BCIS, assess its associated symptoms, and identify potential risk factors that contribute to its occurrence. In addition, we aim to provide recommendations on the prevention and management of this syndrome.

## Methods

This scoping review was conducted according to the Preferred Reporting Items for Systematic reviews and Meta-Analyses extension for Scoping Reviews (PRISMA-ScR) methodology and guidance developed by PRISMA-ScR.^8^

### Eligibility criteria

The inclusion criteria for this scoping review were as follows: (1) patients undergoing arthroplasty; (2) intraoperative measuring moments related to reaming, nailing, cementation, or prosthesis implantation; (3) outcome measurements described according to BCIS grading score^1^ (hypoxaemia instead of hypoxia was used) or, when this grading system was not used, by noting systemic hypotension, hypoxaemia, or both directly related to the insertion of cement or prostheses; (4) peer-reviewed articles written in English; and (5) to ensure healthcare relevancy, all articles published after 1993 were included. Exclusion criteria were: (1) animal studies; (2) case reports, correspondence, reviews, posters, abstracts, editorials, technical reports, and guidelines; (3) measuring moments not related to reaming, nailing, cementation, or prosthesis implantation; and (4) articles describing transoesophageal echocardiography (TOE) results without haemodynamic or pulmonary evaluation; (5) vertebroplasty, kyphoplasty, or cementoplasty, as this involves a different type of surgery; and (6) in case of double publication, we used the article with the largest sample size.

### Search strategy

To identify appropriate keywords, a limited preliminary search was conducted in the databases PubMed, Cochrane, and Embase. A final search was done in PubMed, Cochrane, and Embase on March 5, 2025. The search strategy conducted in PubMed is shown in Supplementary Appendix 1. After removal of duplications, two authors (KB and MG) independently reviewed titles and abstracts using the screening tool Rayyan Software as a Service (Qatar Computing Research Institute, Cambridge, MA, USA).^9^ Irrelevant studies were removed according to the inclusion and exclusion criteria. Subsequently, the same two reviewers (KB and MG) screened the available full-texts. Any disagreements between reviewers were resolved through discussion. In case of uncertainty for inclusion, consensus was reached by discussion with other researchers (PN, MS, and SS). Reference lists of the included studies were checked to identify relevant studies eligible for inclusion. A risk-of-bias assessment was not conducted for the included studies, consistent with standard practice in scoping reviews.

### Data charting

A study-specific, data extraction form was developed in Excel (Microsoft Corporation, Redmond, WA, USA) using an iterative process continually updating the form. Two reviewers (KB and MG) independently extracted the study characteristics and outcomes. Figures were made in Excel (Microsoft Corporation). The references were managed in Mendeley (Mendeley Desktop, Amsterdam, The Netherlands).

## Results

**Fig 1:**
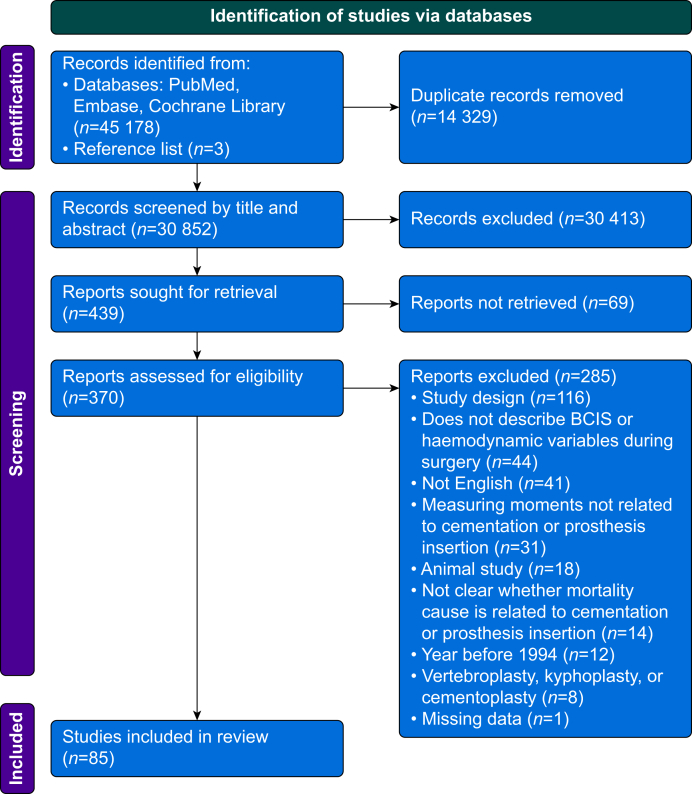
Flow chart for selection of included studies. BCIS, bone cement implantation syndrome.

The initial search yielded 45 178 studies, of which 30 852 studies were eligible for screening after duplication removal. After title and abstract screening, 30 413 studies were excluded. The full-text articles were assessed for eligibility, which led to 85 included studies. A flow chart is provided in Figure 1.

### Types of included studies

The included studies comprised 29 RCTs (34.1%),^10^, ^11^, ^12^, ^13^, ^14^, ^15^, ^16^, ^17^, ^18^, ^19^, ^20^, ^21^, ^22^, ^23^, ^24^, ^25^, ^26^, ^27^, ^28^, ^29^, ^30^, ^31^, ^32^, ^33^, ^34^, ^35^, ^36^, ^37^, ^38^ 29 prospective studies (34.1%),^39^, ^40^, ^41^, ^42^, ^43^, ^44^, ^45^, ^46^, ^47^, ^48^, ^49^, ^50^, ^51^, ^52^, ^53^, ^54^, ^55^, ^56^, ^57^, ^58^, ^59^, ^60^, ^61^, ^62^, ^63^, ^64^, ^65^, ^66^, ^67^ 24 retrospective studies (28.2%),^2^^,^^68^, ^69^, ^70^, ^71^, ^72^, ^73^, ^74^, ^75^, ^76^, ^77^, ^78^, ^79^, ^80^, ^81^, ^82^, ^83^, ^84^, ^85^, ^86^, ^87^, ^88^, ^89^, ^90^ two surveys (2.4%),^91^^,^^92^ and one surveillance study (1.2%).^93^ Supplementary Appendix 2 provides an overview with study characteristics and outcomes of the included studies.

A total of 41 824 participants and 51 541 joints were included. Owing to double publication, patients who underwent cemented arthroplasty in the study by Olsen and colleagues^79^ were excluded (*n*=986), and only data from uncemented arthroplasties were included. Table 1 presents a summary of the number of articles categorised by surgery type, diagnosis related to the surgery, fixation method, and anaesthesia technique. In all, but four, studies haemodynamic or pulmonary effects were measured.^33^^,^^73^^,^^91^^,^^93^ Intraoperative TOE was performed in 27 studies.^11^, ^12^, ^13^^,^^15^^,^^19^^,^^23^^,^^26^^,^^28^, ^29^, ^30^, ^31^, ^32^^,^^34^^,^^38^, ^39^, ^40^^,^^43^, ^44^, ^45^^,^^50^^,^^52^^,^^53^^,^^59^, ^60^, ^61^^,^^63^^,^^65^ Fourteen studies compared cemented with uncemented arthroplasty.^11^^,^^12^^,^^17^^,^^22^^,^^32^^,^^40^^,^^41^^,^^43^^,^^48^^,^^53^^,^^54^^,^^56^^,^^67^^,^^79^

**Table 1:** Study characteristics. ∗Single observations are registered in multiple categories. Data are presented as *n* (%).

	No. of articles (*N*=85)
**Type of surgery∗**	
Hip arthroplasty	63 (74.1)
Knee arthroplasty	14 (16.5)
Intramedullary nailing	11 (12.9)
Shoulder arthroplasty	1 (1.2)
**Diagnosis for surgery∗**	
Trauma	44 (51.8)
Nontraumatic diagnosis	22 (25.9)
Pathological (impending) fracture	9 (10.6)
Malignant disease	7 (8.2)
Not reported	19 (22.4)
**Fixation method∗**	
Cemented	54 (63.5)
Cemented and uncemented	20 (23.5)
Uncemented	9 (10.6)
Not reported	2 (2.4)
**Anaesthesia method∗**	
General	48 (56.5)
Spinal	15 (17.6)
General–epidural	7 (8.2)
General–regional	6 (7.1)
Epidural	5 (5.9)
Regional	3 (3.5)
Spinal–epidural	2 (2.4)
Not reported	17 (20.0)

Based on the research questions and through the iterative process of data collection, five domains were identified across the eligible studies: (1) incidence; (2) haemodynamic and pulmonary function; (3) embolic events; (4) risk factors; and (5) prevention and treatment.

### Incidence

Twenty-one studies have been published describing the incidence of BCIS grade 1 and 2 according to the classification.^2^^,^^20^^,^^34^^,^^54^^,^^55^^,^^64^^,^^66^^,^^67^^,^^69^, ^70^, ^71^^,^^75^^,^^77^, ^78^, ^79^^,^^84^^,^^86^, ^87^, ^88^, ^89^, ^90^ Another five studies did not use this classification and described the incidence of systemic hypotension, hypoxaemia, or both related to cementation or prosthesis insertion.^27^^,^^72^^,^^76^^,^^81^^,^^83^ They defined hypotension as a decrease in mean arterial pressure (MAP) >15 mm Hg, a decrease in SBP >30 mm Hg or decrease >30% from the initial baseline systolic average. However, because of the different classifications used, including these five articles would cause nonuniform conclusions. We therefore chose not to describe these studies in this part of the review.

Table 2 shows the reported incidences of BCIS. Sixteen studies included patients receiving a cemented hip arthroplasty for a femoral neck fracture or a nontraumatic disease and described an overall incidence (grade 1–3) ranging from 15.4% to 46.7%,^2^^,^^20^^,^^54^^,^^66^^,^^67^^,^^69^^,^^71^^,^^75^^,^^77^^,^^78^^,^^87^^,^^90^ with three outliers at 4.2%,^89^ 72.2%,^70^ and 100%^55^^,^^64^ (Table 2). Table 2 shows the incidence for patients receiving uncemented hip arthroplasty ranging from 0.0% to 21.8%.^54^^,^^55^^,^^67^^,^^79^

**Table 2:** Incidence of bone cement implantation syndrome. THA, total hip arthroplasty.

Author (year)	Intervention group(s)	No. of participants	Overall (%)	Grade 1 (%)	Grade 2 (%)	Grade 3 (%)
**Cemented hip arthroplasty for trauma or nontraumatic diagnosis**						
Bhadani and colleagues^66^ (2024)	THA or hemiarthroplasty	72	20.8	18.1	0.0	2.8
Bökeler and colleagues^69^ (2022)	Hemiarthroplasty, third-generation cementing technology	49	17.4	**–**	**–**	**–**
	Hemiarthroplasty, second-generation cementing technology	43	25.6	**–**	**–**	**–**
Chen and colleagues^70^ (2016)	Hemiarthroplasty	1210	72.2	42.2	21.0	9.0
Chulsomlee and colleagues^71^ (2023)	THA	128	32.8	26.6	6.3	2.3
Fernandez and colleagues^67^ (2025)	Hemiarthroplasty	46	15.4	15.4	0.0	0.0
García-Mansilla and colleagues^75^ (2023)	THA	273	18.7	13.6	5.1	0.0
Jaffe and colleagues^77^ (2022)	Hemiarthroplasty	69	35.0	25.6	10.1	0.0
Kaufmann and colleagues^20^ (2018)	THA, goal-directed therapy	45	37.8	37.8	0.0	0.0
	THA, conventional therapy	45	46.7	31.1	15.6	0.0
Miyamoto and colleagues^54^ (2017)	Hemiarthroplasty	86	30.2	25.6	4.7	0.0
Motobe and colleagues^55^ (2004)	THA or hemiarthroplasty	16	100	–	–	–
Olsen and colleagues^78^ (2014)	Hemiarthroplasty	1016	28.0	21.0	5.1	1.7
Rassir and colleagues^2^ (2021)	Hemiarthroplasty	915	31.0	22.0	8.0	0.4
	THA	677	24.0	19.0	5.0	0.0
Ukaj and colleagues^64^ (2021)	THA, age <66.5 yr	13	23.1	23.1	0.0	0.0
	THA, age >66.5 yr	12	100	83.3	16.7	0.0
Weingärtner and colleagues^87^ (2021)	Hemiarthroplasty	208	37.0	25.5	8.2	3.4
Yuenyongviwat and colleagues^89^ (2024)	Hemiarthroplasty	311	4.2	4.2	0.0	0.0
Zastrow and colleagues^90^ (2025)	Hemiarthroplasty	137	42.3	35.0	6.6	0.7
**Uncemented hip arthroplasty for trauma or nontraumatic diagnosis**						
Fernandez and colleagues^67^ (2025)	Hemiarthroplasty	46	5.0	0.0	0.0	0.0
Miyamoto and colleagues^54^ (2017)	Hemiarthroplasty	78	21.8	17.9	3.8	0.0
Motobe and colleagues^55^ (2004)	THA or hemiarthroplasty	19	0.0	**–**	–	–
Olsen and colleagues^79^ (2020)	Hemiarthroplasty	109	17.0	17.0	0.0	0.0
**Cemented arthroplasty for malignant disease**						
Rao and colleagues^84^ (2022)	Hip or knee arthroplasty	67	46.3	34.3	11.9	0.0
Schwarzkopf and colleagues^86^ (2019)	THA or hemiarthroplasty	374	74.0	62.5	11.0	0.5
Singh and colleagues^34^ (2018)	THA (vacuum technique)	16	31.3	**–**	31.3	0.0
	THA (conventional technique)	16	75.0	**–**	75.0	0.0
Yang and colleagues^88^ (2021)	Tumour surgery	88	26.1	25.6	4.5	0.0

Four studies focused on cemented arthroplasty in patients with a malignant disease (Table 2). The reported incidences ranged from 26.1% to 75.0%.^34^^,^^84^^,^^86^^,^^88^ Two studies described patients undergoing cemented total knee arthroplasty and reported an overall incidence of BCIS of 27.5%^2^ and 70.7%.^14^ One study investigating patients receiving cemented shoulder arthroplasty reported an incidence of 16.2%.^2^ No studies reported the incidence of BCIS in uncemented total knee or shoulder arthroplasty.

Twenty-seven studies described cardiopulmonary resuscitation after cementation or prosthesis insertion, thus BCIS grade 3.^2^^,^^14^^,^^20^^,^^27^^,^^34^^,^^54^^,^^55^^,^^64^^,^^66^^,^^67^^,^^69^, ^70^, ^71^, ^72^^,^^75^, ^76^, ^77^, ^78^, ^79^^,^^81^^,^^83^^,^^84^^,^^86^, ^87^, ^88^, ^89^, ^90^ In patients receiving a cemented hip arthroplasty for a femoral neck fracture or a nontraumatic disease, the incidence ranged from 0.0% to 3.4%,^2^^,^^14^^,^^20^^,^^54^^,^^55^^,^^64^^,^^66^^,^^67^^,^^69^^,^^71^^,^^75^^,^^77^^,^^78^^,^^87^^,^^89^^,^^90^ with one outlier of 9.0%.^70^ In cemented hip arthroplasty for a malignant disease, the incidence was from 0.0% to 0.5%,^27^^,^^34^^,^^76^^,^^81^^,^^83^^,^^84^^,^^86^^,^^88^ and in one study 5.3%.^72^ Grade 3 was not reported in cemented knee arthroplasty, or in uncemented hip, shoulder, or knee arthroplasty. Moreover, one article described intraoperative deaths during hip arthroplasty, although they did not include patients in whom resuscitation was successful. The authors reported an overall intraoperative mortality of 0.05% for a patient undergoing a total hip arthroplasty and 0.17% for a patient undergoing a hemiarthroplasty.^80^

In all study groups (hip, knee, and shoulder arthroplasty), BCIS grade 1 was more common than grades 2 or 3. In uncemented arthroplasty, BCIS grade 3 was not observed, and grade 2 was rare. Moreover, the occurrence of BCIS grade 2 or 3 showed a significant increase in mortality during hospital admission, within 30 days and 1 yr postsurgery.^2^^,^^78^^,^^87^

### Haemodynamic and pulmonary function

BCIS is characterised by hypoxaemia (SpO_2_ <94%), hypotension (a decrease in SBP >20%), or both. Various other pulmonary and haemodynamic functions that were plausibly affected by cementing, or prosthesis or nail insertion were investigated (Table 3).

**Table 3:** The investigated pulmonary and haemodynamic functions that could be influenced by cementing, or prosthesis or nail insertion. ETCO_2_, end-tidal carbon dioxide; HR, heart rate; MAP, mean arterial pressure; MPAP, mean pulmonary artery pressure; PaO_2_, partial oxygen pressure.

Variable	Mean decrease	No. of articles	No change	No. of articles	Mean increase	No. of articles
HR (n=25)	< −10 beats min^−1^	0	−10 to 10 beats min^−1^	19	> 10 beats min^−1^	6
ETCO_2_ (n=8)	< −0.7 kPa	1	−0.7 to 0.7 kPa	7	> 0.7 kPa	0
PaO_2_ (n=11)	< −4 kPa	3	−4 to 4 kPa	8	> 4 mmHg	0
MAP (n=20)	< −10 mmHg	4	−10 to 10 mmHg	13	> 10 mmHg	3
MPAP (n=4)	< −5 mmHg	0	−5 to 5 mmHg	2	> 5 mmHg	2

#### Heart rate

All of the 25 cemented arthroplasty studies reported an increase in mean heart rate.^14^^,^^16^^,^^17^^,^^19^^,^^20^^,^^25^^,^^28^^,^^31^^,^^35^, ^36^, ^37^, ^38^^,^^41^^,^^48^^,^^49^^,^^51^^,^^56^^,^^58^, ^59^, ^60^^,^^62^^,^^64^, ^65^, ^66^^,^^82^ However, in six studies the mean increase exceeded more than 10 beats min^−1^.^19^^,^^36^^,^^41^^,^^62^^,^^64^^,^^82^

#### End-tidal carbon dioxide

The end-tidal carbon dioxide (ETCO_2_) was measured in eight studies performing cemented arthroplasty.^19^^,^^23^^,^^28^^,^^29^^,^^32^^,^^56^^,^^59^^,^^60^ All of these studies showed a decrease, but only one study reported a mean decrease of more than 0.7 kPa.^19^

### Partial pressure of arterial oxygen

Eleven studies measured partial pressure of arterial oxygen (Pao_2_) in cemented arthroplasty,^16^^,^^17^^,^^19^^,^^35^^,^^38^^,^^48^, ^49^, ^50^, ^51^^,^^53^^,^^56^ of which three studies^16^^,^^17^^,^^50^ showed a mean decrease of more than 4 kPa. Furthermore, three studies reported that Pao_2_ changes were correlated with the severity of embolic phenomena. They observed no significant Pao_2_ change during embolic events grade 0 or 1, whereas during embolic events grade 2 or 3 a significant change was observed.^43^^,^^44^^,^^63^

Moreover, various studies have investigated systemic vascular resistance index, stroke volume, cardiac output, cardiac index, central venous pressure, pulmonary vascular resistance index, and right ventricular ejection fraction.^16^^,^^17^^,^^28^^,^^36^^,^^41^^,^^46^^,^^47^^,^^49^^,^^53^^,^^58^, ^59^, ^60^, ^61^^,^^65^ However, conflicting results were presented.

### Embolic events

The passage of echogenic material in the right cavities of the heart was studied using TOE during intramedullary nailing, total hip arthroplasty, or hemiarthroplasty in 16 studies^11^, ^12^, ^13^^,^^23^^,^^29^, ^30^, ^31^, ^32^^,^^39^^,^^40^^,^^43^^,^^44^^,^^50^^,^^53^^,^^61^^,^^65^ and during total knee arthroplasty in seven studies.^19^^,^^26^^,^^28^^,^^38^^,^^52^^,^^59^^,^^60^ Five studies compared the quantity of echogenic material in the right side of the heart during cemented and uncemented surgery.^11^^,^^12^^,^^32^^,^^40^^,^^43^ All five studies showed that cemented arthroplasty was associated with more prolonged and more severe echogenic events than uncemented arthroplasty. To study the composition of the debris, seven studies^11^^,^^12^^,^^21^^,^^26^^,^^48^^,^^52^^,^^59^ aspirated blood from the right atrium using a central venous, pulmonary artery, or antecubital catheter, and one study aspirated blood from the femoral vein using a femoral venous sheath. They showed a composition potentially consisting of fat,^11^^,^^12^^,^^21^^,^^26^^,^^48^^,^^52^ bone marrow,^11^^,^^12^ fresh thrombus,^59^ air,^12^^,^^26^ aggregates or platelets,^11^ and fibrin.^12^

### Risk factors

**Fig 2:**
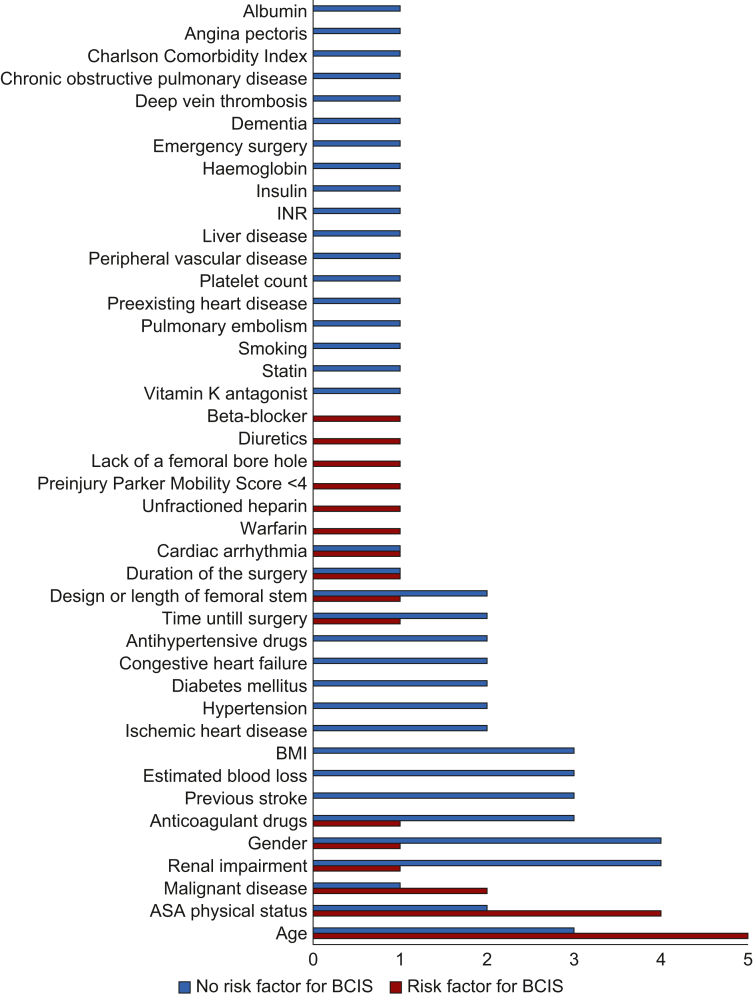
Number of times risk factors were analysed and identified in a study. BCIS, bone cement implantation syndrome; INR, international normalized ratio.

Identification of potential risk factors for the development of BCIS with either univariate or multivariate analysis was done in 11 studies (Fig. 2).^2^^,^^70^^,^^71^^,^^75^^,^^78^^,^^84^^,^^86^, ^87^, ^88^, ^89^, ^90^ When two or more studies suggested that a certain factor was a risk factor, it was considered as such in this review. Therefore, age, ASA 3 or 4, and primary lung cancer or lung metastasis are regarded as potential risk factors. An additional 10 studies described risk factors, but did not perform statistical analyses.^29^^,^^33^^,^^52^^,^^58^^,^^63^^,^^64^^,^^74^^,^^76^^,^^77^^,^^80^

Age was an important risk factor for the development of BCIS in five studies,^2^^,^^70^^,^^71^^,^^86^^,^^87^ whereas three studies reported age as not being a risk factor.^75^^,^^78^^,^^88^ Moreover, one study reported age as a risk factor for the occurrence of embolic events in the right atrium,^52^ whereas another study did not find this correlation.^63^ Four studies reported ASA physical status 3 or 4 as a potential risk factor,^2^^,^^70^^,^^78^^,^^87^ whereas two studies did not find this correlation.^71^^,^^75^ Two studies identified primary lung cancer or lung metastasis as a potential risk factor.^86^^,^^88^ In addition, three articles reported more haemodynamic and pulmonary complications in patients with malignant disease, but statistical analyses were not performed.^61^^,^^68^^,^^76^

### Prevention and treatment

Prevention of BCIS, haemodynamic or pulmonary instability, or the occurrence of embolic events were investigated in 33 studies during hip or knee surgery.^10^^,^^13^, ^14^, ^15^, ^16^^,^^18^^,^^20^^,^^21^^,^^23^, ^24^, ^25^, ^26^^,^^28^, ^29^, ^30^, ^31^, ^32^, ^33^, ^34^, ^35^, ^36^, ^37^, ^38^^,^^44^^,^^45^^,^^57^^,^^63^^,^^69^, ^70^, ^71^^,^^75^^,^^82^^,^^84^

#### Surgical developments

Various surgical techniques were described in 22 studies.^13^^,^^15^^,^^18^^,^^21^^,^^23^^,^^26^^,^^28^, ^29^, ^30^, ^31^, ^32^, ^33^, ^34^, ^35^^,^^38^^,^^44^^,^^45^^,^^57^^,^^63^^,^^69^^,^^71^^,^^82^ The third-generation, decompression, or suctioning technique potentially causes fewer embolic events and haemodynamic or pulmonary disturbances.^23^^,^^29^, ^30^, ^31^, ^32^^,^^34^^,^^35^^,^^45^^,^^63^^,^^69^ Medullary lavage has also been associated with a decrease in embolic events and haemodynamic or pulmonary disturbances.^13^^,^^38^^,^^82^ In addition, reaming systems showed a reduction of embolic events, but no correlation with any change in physiologic variables could be seen.^15^^,^^18^^,^^44^^,^^57^

#### Anaesthesia methods

Four studies investigated the association between anaesthesia and the incidence of BCIS, or haemodynamic or pulmonary instability in patients during hip or knee arthroplasty for femoral fractures.^10^^,^^70^^,^^84^^,^^90^ First, it has been reported that the use of general anaesthesia alone was associated with a 5.8-fold increased risk of developing BCIS compared with patients who also received neuraxial anaesthesia.^84^ This finding aligns with a study that showed a higher incidence of BCIS in patients who received general anaesthesia (55.3%) or general combined with regional anaesthesia (44.8%) compared with those who received neuraxial anaesthesia (18.8%).^90^ In contrast, another study observed less haemodynamic instability during general anaesthesia compared with spinal anaesthesia.^10^ Lastly, a study compared spinal anaesthesia with combined spinal–epidural anaesthesia and showed a comparable incidence of BCIS in both groups.^70^

#### Fluid administration

Two studies investigated the effect of fluid therapy on the incidence of BCIS in patients undergoing cemented hip or knee arthroplasty.^14^^,^^20^ Kaufmann and colleagues^20^ described a reduction in the prevalence of BCIS grade 2 as a result of goal-directed therapy during total hip arthroplasty. However, a reduction of BCIS in total, grade 1–3, was not observed. The study by Dumanli Özcan and colleagues^14^ showed a significant reduction of BCIS grade 1 when patients received a 8 ml kg^−1^ bolus of hydroxyethyl starch 20 min before or at the time of cementation compared with patients who received infusions of 8 ml kg^−1^ h^−1^ sodium chloride during the anaesthesia management.

#### Medical intervention

Six studies described the effect of medication on the prevention or treatment of BCIS, or any haemodynamic or pulmonary effects.^16^^,^^24^^,^^36^^,^^37^^,^^75^^,^^94^ The first study reported that patients receiving goal-directed therapy combined with phenylephrine showed less haemodynamic and pulmonary disturbances compared with patients only receiving goal-directed therapy.^36^ The second study showed that prophylactic i.v. administration of 45.5 mg pheniramine maleate (histamine-1 receptor antagonist) and 8 mg dexamethasone mixture significantly decreased the dose of adrenaline and atropine used after cementation, and the peripheral oxygen saturation values were more stable compared with patients receiving normal saline. Therefore, they suggested that it could minimise the clinical symptoms of BCIS.^37^ Conversely, the third study did not find a significant difference for the occurrence of BCIS between patients receiving prophylactic i.v. administration of a mixture of 2 mg clemastine (histamine-1 receptor antagonist) and 200 mg cimetidine (histamine-2 receptor antagonist), and the control group.^24^ In the fourth study, prophylactic administration of 10 IU^−1^ unfractionated heparin intraoperative just before cementation increased the incidence of BCIS events compared with patients not receiving the unfractionated heparin. In patients who received unfractionated heparin, the incidence of BCIS was 35.1%, whereas in patients who did not receive unfractionated heparin, the incidence was 2.9%. They hypothesised that this could be explained by anticoagulants causing plaque haemorrhage which results in cholesterol microembolisation.^75^^,^^95^ The fifth and the sixth study showed that inhalation of prostacyclin (prostaglandin I_2_) did not give any haemodynamic or pulmonary effects.^16^^,^^94^

#### Predictive nursing model

The predictive nursing model is a design to identify and anticipate potential issues, enabling nurses to take preventive actions and reduce risks proactively. Li and colleagues^25^ showed that implementing this model minimised the haemodynamic compromises during BCIS.

#### Treatment

A solitary study suggested a treatment option for BCIS. The authors advocated the utilisation of temporary extracorporeal membrane oxygenation in patients who do not respond to standard management procedures and persist with hypoxaemia.^85^

## Discussion

In this review we aimed to identify the incidence, clinical presentation, and risk factors of BCIS. BCIS is frequently observed in cemented and uncemented procedures. However, BCIS grade 3 remains rare in cemented arthroplasty and has not been reported in uncemented procedures. Moreover, BCIS has been observed not only in hip arthroplasty but also during knee and shoulder procedures. The overall incidence of BCIS in patients without malignancy during cemented hip arthroplasty was from 15.4% to 46.7%,^2^^,^^20^^,^^54^^,^^66^^,^^67^^,^^69^^,^^71^^,^^75^^,^^77^^,^^78^^,^^87^^,^^90^ during cemented knee arthroplasty was from 25.7%^2^ to 70.7%,^14^ and during cemented shoulder arthroplasty was 16.2%.^2^ In uncemented hip arthroplasty, the incidence ranged from 0.0% to 21.8%.^54^^,^^55^^,^^67^^,^^79^ Although several pulmonary and haemodynamic functions were investigated, only heart rate increased during the occurrence of BCIS. Risk factors for BCIS were advanced age, ASA physical status 3 or 4, and primary lung cancer or lung metastasis.

The results of this study show that BCIS is a commonly observed phenomenon in both cemented and uncemented arthroplasty. An explanation might be the fact that intramedullary pressure also increases during uncemented arthroplasty.^96^^,^^97^ This increased pressure can result in embolic debris, albeit to a less prolonged and severe degree than in cemented arthroplasty.^11^^,^^12^^,^^32^^,^^40^^,^^43^ Because BCIS is observed in both fixation methods, we conclude that the occurrence of BCIS is not dependent on the use of cement. Still, BCIS appears to be more frequent and severe in cemented than in uncemented arthroplasty. Based on this, we suggest that the severity of BCIS does have an association with the fixation method, as only BCIS grades 1 and 2 have been reported in uncemented arthroplasty, with grade 2 occurring infrequently and grade 3 not observed at all. However, it is worth noting that the number of participants in the included studies performing uncemented arthroplasty is limited. Therefore, the absence of BCIS grade 3 in uncemented procedures may reflect either a true lack of severe cases, or it may be owing to small sample sizes. More research on uncemented arthroplasty is needed.

The aetiology of BCIS is unknown, although five theories have been proposed. The first theory posits that methyl methacrylate (MMA) monomers may induce vasodilatation. However, studies showed that plasma MMA concentrations are too low to cause systemic effects.^98^^,^^99^ Additionally, a recent literature review was conducted to assess whether sufficient evidence exists to support a causal relationship between cement use and BCIS. The findings indicate that the available evidence is currently inadequate to conclusively establish such a link.^100^ The second theory suggests an anaphylactic reaction, leading to an increase in plasma histamine. The third theory implicates the activation of complement factors C3a and C5a, which are mediators of vasoconstriction and bronchoconstriction. The fourth theory involves the release of embolic debris. The fifth theory suggests that these pathophysiological cascades occur simultaneously.^1^ However, little research about the first three theories has been performed with contradictory results as opposed to the embolic theory, which has been more frequently studied. In several postmortem animal studies, the debris retrieved from the right atrium was analysed and identified predominantly as fat,^11^^,^^12^^,^^21^^,^^26^^,^^39^^,^^48^^,^^52^^,^^97^^,^^101^ air,^12^^,^^26^ bone marrow,^11^^,^^12^^,^^102^ fresh thrombus,^59^ aggregates of platelets,^11^ and fibrin.^12^ Although the studies that analysed debris included participants receiving both cemented and uncemented arthroplasty, none of the studies described the presence of cement particles.^94^^,^^97^^,^^101^^,^^103^, ^104^, ^105^, ^106^ In postmortem studies of patients who died minutes after cemented total hip arthroplasty, histological examination showed disseminated microembolisation. Fat particles could be seen in the blood vessels of the lungs, liver, brain, heart, and kidneys.^73^^,^^80^^,^^107^, ^108^, ^109^ One study performed postmortem CT imaging on patients who had undergone cemented arthroplasty an average of 22 months prior. Pulmonary cement embolisms were reported in 46.3% of the patients, indicating that these embolisms can be long-lasting and not related to death.^110^

The embolisation during surgery can be seen and classified using a TOE. However, it is challenging to distinguish between air and fat embolism. An animal study demonstrated that although pigs injected with >0.5 mg kg^−1^ of air showed an echogenic pattern grade 3 (emboli >5 mm in diameter), this pattern was not produced by fat injections.^111^ Total hip arthroplasty is reported as a high-risk procedure for the development of venous air embolisms.^112^ This raises the question whether the impact of air embolism has been underestimated. Further research is needed to clarify whether the embolic signals observed on TOE are predominantly caused by air rather than fat and bone marrow embolism.

A survey among orthopaedic surgeons and anaesthesiologists showed that BCIS was six times as frequent in traumatology as in scheduled surgery.^91^ In addition, in a patient undergoing hip arthroplasty for a fracture, the incidence of death was six times higher when compared with patients undergoing arthroplasty not related to a fracture.^80^ There are two possible explanations for this observation. First, age is considered a risk factor for BCIS and most traumatology patients are relatively old.^113^^,^^114^ Second, these patients are vulnerable to an increased bone porosity.^115^ Although this increased porosity causes the increase in intramedullary pressure to be less pronounced in older patients than younger patients, it also leads to more embolisation during pressurisation of the medullary canal. This correlation between age and the total amount of embolism is also shown in studies that performed TOE.^43^^,^^52^ In addition, another study found that older patients had more pronounced hypoxia than younger patients.^64^ Moreover, most studies agree that the more severe the embolisation, the greater the deterioration in haemodynamic and pulmonary functions, leading to an increased risk of BCIS.^11^^,^^13^^,^^19^^,^^23^^,^^43^, ^44^, ^45^^,^^61^^,^^63^

To identify more variables for detecting BCIS, studies examined factors such as the heart rate, cardiac output, ETCO_2_, and mean pulmonary artery pressure, as these serve as surrogate markers for the pulmonary vascular obstruction and hypertension observed in pulmonary embolisms. However, there is no convincing evidence to support the use of these hemodynamic and respiratory variables for detecting BCIS. Therefore, we do not recommend measuring any variables other than SBP and SpO_2_.

Optimising prevention and management strategies for BCIS requires consideration of both surgical technique and anaesthetic approach. The choice between cemented and uncemented arthroplasty should be guided by the incidence and severity of BCIS associated with each fixation method, and patient-specific risk factors. In cases of uncertainty or when treating high-risk patients, consultation with the anaesthesiologist is advisable. When deciding to perform cemented arthroplasty, the use of third-generation or decompression technique has been shown to reduce embolic events and decrease haemodynamic and pulmonary complications, making these techniques preferable.^23^^,^^29^^,^^31^^,^^32^^,^^34^^,^^35^^,^^63^^,^^69^ Furthermore, the potential role of anaesthesia in preventing BCIS remains uncertain. Existing evidence indicates a slight preference for neuraxial anaesthesia, or a combination of general and neuraxial anaesthesia, over general anaesthesia alone.^10^^,^^84^^,^^90^^,^^116^ However, studies comparing spinal and general anaesthetic approaches have produced conflicting results regarding mortality outcomes.^117^, ^118^, ^119^, ^120^, ^121^, ^122^

It is well-established that poor or absent communication is a common contributor to adverse surgical outcomes.^123^^,^^124^ Therefore, closed-loop communication between the surgical and anaesthetic teams is important for promptly identifying and addressing any signs of BCIS. During both cemented and uncemented arthroplasty, the risk of embolisation is highest during reaming,^19^^,^^40^ prosthesis insertion or cementation,^12^^,^^13^^,^^19^^,^^23^^,^^29^, ^30^, ^31^, ^32^^,^^40^^,^^43^^,^^44^^,^^50^^,^^53^^,^^65^ and hip relocation.^12^^,^^23^^,^^31^^,^^32^^,^^43^^,^^44^ As a result, anaesthesiologists should remain especially vigilant during these stages. Furthermore, it is recommended to maintain normovolaemia before and throughout surgery, and to administer 100% oxygen during the high-risk moments. Upon suspicion of BCIS, immediate intervention is required, including resuscitation based on standard protocols with aggressive fluid resuscitation, and administration of vasopressors and inotropes.

Research on effective treatments of BCIS is still limited, although several studies have been conducted to stabilise blood pressure and reduce the use of vasopressor drugs during surgery. In two recent RCTs, older patients undergoing hip or knee arthroplasty under spinal anaesthesia were given either 5 mg or 10 mg of midodrine or a placebo. Both studies concluded that midodrine decreased the incidence of hypotension and reduced the need of vasopressor requirements.^125^^,^^126^ Further research is required to determine whether this oral vasopressor could also be effective in preventing BCIS.

In 2009 Donaldson and colleagues^1^ proposed a definition and classification system for BCIS. However, because BCIS has been observed in both cemented and uncemented arthroplasty, the term ‘bone cement implantation syndrome' may be an inaccurate term.^100^ Further research on uncemented procedures is required before reconsidering the terminology.

The proposed definition does not specify a timeframe within which symptoms should manifest. This may have contributed to heterogeneity across studies. We suggest adding a timeframe to the definition, stipulating that hypotension, hypoxaemia, or both should occur from reaming, prosthesis implantation, or cementation up until skin closure. Beyond this point, various anaesthetic interventions could influence the haemodynamic and pulmonary functions. Additionally, the most widely used definition for intraoperative hypotension is a MAP <65 mm Hg, based on clear evidence that organ injury can occur when MAP decreases below this measure, with the risk increasing significantly at MAP values <55 mm Hg.^127^, ^128^, ^129^, ^130^, ^131^ However, in the context of BCIS, the relative change in MAP is particularly important for identifying the syndrome. As patients may already have a MAP ≤65 mm Hg before reaming, prosthesis implantation, or cementation, it is crucial to consider the magnitude of change rather than relying solely on absolute values. Therefore, we propose incorporating a relative MAP decrease in the classification: a 15% decrease for BCIS grade 1 and a 30% decrease for BCIS grade 2.

Grade 1: moderate hypoxaemia (SpO2 <94%) or hypotension (decrease in SBP >20% and <40% or decrease in MAP >15% and <30%).

Grade 2: severe hypoxaemia (SpO2 <88%) or hypotension (decrease in SBP >40% or decrease in MAP >30%) or unexpected loss of consciousness.

Grade 3: cardiovascular collapse requiring cardiopulmonary resuscitation.

### Strengths and limitations

The strength of this study lies in its comprehensive search strategy, which minimises the risk of missing relevant articles. However, it is still possible that some eligible papers were overlooked. A limitation to consider is the small number of participants in the included studies, which should be taken into account when interpreting the results of this review. In addition, the studies are heterogeneous, making it difficult to compare them and draw conclusions.

### Conclusions

*Bone cement implantation syndrome* is a common phenomenon in cemented and uncemented arthroplasty, although less pronounced in uncemented arthroplasty. Therefore, we conclude that the occurrence of *bone cement implantation syndrome* is not dependent on the use of cement. However, the severity does have an association with the fixation method. Additionally, BCIS occurs not only in hip arthroplasty but also during knee and shoulder procedures. Risk factors include advanced age, ASA physical status 3 or 4, and primary lung cancer or lung metastasis. More research is needed to establish effective prevention and treatment strategies.

## Authors’ contributions

Study design data collection: KEB, MG, INS

Writing of manuscript: KEB

Table layout design: KEB

Valuable feedback during the writing process: MG, INS, SS, MAHS, PAN

Approval of final manuscript version: all authors

## Declaration of interest

The authors declare no competing interest.
