# Supplementary material for: Bone cement implantation syndrome: a scoping review
Source: Br J Anaesth. 2025 Jul 8;135(4):1038–50. doi: 10.1016/j.bja.2025.05.041 (PMC12674061; doi:10.1016/j.bja.2025.05.041)
Supplement: Multimedia component 1 [file mmc1.docx]

Supplementary Appendices

Supplementary Appendix 1: Search

Pubmed: 5 March 2025

(((((((((((("Arthroplasty"[Mesh] AND "Embolism"[Mesh]) OR (''Intraoperative Complications''[mesh] AND "Bone Cements"[Mesh])) OR (("Hip Fractures"[Mesh] OR "Fracture Fixation, Intramedullary"[Mesh] OR "Arthroplasty"[Mesh] OR "Orthopedic Procedures"[Majr:NoExp] OR "Femur/surgery"[Majr] OR "Knee Prosthesis"[Mesh]) AND ("Pulmonary Embolism"[Mesh] OR "Hemodynamics"[Mesh] OR "Heart Arrest"[Mesh] OR pulmonary embolism[tiab] OR "Bone Cements"[Mesh] OR ''Polymethyl Methacrylate''[mesh] OR "Methylmethacrylate"[Mesh]))) OR ("Heart Arrest"[Mesh] AND ("Femoral Fractures"[Mesh] OR "Knee Prosthesis"[Mesh]))) OR (cement implantation syndrome*[tiab])) OR ((hip arthroplasty’[tiab] OR knee arthroplasty'[tiab]) AND cement*[tiab])) OR ((pulmonary complication*[tiab] OR pulmonary impairment[tiab]) AND (femoral fracture*[tiab] OR femur fracture*[tiab]))) OR ((intramedullary nail*[tiab] OR intramedullary surgery[tiab] OR femoral nailing[tiab]) AND (emboli*[tiab] OR bone marrow fat intravasation[tiab]))) OR ((Cemented[tiab] OR Cement[tiab]) AND (Embolism[tiab] OR embolization*[tiab]))) OR ((arthroplasty[tiab] AND hypotension[tiab]))) OR (cemented[tiab] AND mortality[tiab])) OR ((hypotension[tiab] OR blood pressure[tiab]) AND (cement[tiab] OR cemented[tiab] OR methylmethacrylate[tiab]))) OR ((hip joint[tiab] AND hypotension[tiab])) OR ((pulmonary embolism[tiab] AND hip arthroplasty[tiab])) AND ("1990/01/01"[Date - Publication] : "2025/03/05"[Date - Publication])

Supplementary Appendix 2: Overview with study characteristics and outcomes of the included studies. MAP, mean arterial pressure; TOE, transoesophageal echocardiography; THA, total hip arthroplasty; BCIS, bone cement implantation syndrome; ASA, American Society of Anaesthesiologists; CO, cardiac output; SV, stroke volume; HR, heart rate; MPAP, mean pulmonary arterial pressure; SBP, systolic blood pressure; ECMO, Extracorporeal Membrane Oxygenation.

| **Author (year)** | **Country** | **Study design** | **Objectives** | **Intervention groups** | **Diagnosis for surgery** | **Fixation method** | **Method of anaesthesia** | **Number of participants (and number of joints)** | **Measurements** | **Main results** |
| --- | --- | --- | --- | --- | --- | --- | --- | --- | --- | --- |
| Abdelrahman  and colleagues (2023)^1^ | Egypt | RCT | 1. To compare the impact of spinal anaesthesia on elderly patients undergoing cemented hip arthroplasty operations versus general anaesthesia in combination with femoral nerve block on MAP | Hip arthroplasty • General anaesthesia combined with ultra-sound guided femoral nerve block • Spinal anaesthesia | • Femoral fracture • Other | Cemented | • General-regional • Spinal | 60 | • Haemodynamic and pulmonary monitoring | • A significant decrease was seen in MAP after cementation in spinal anaesthesia group • General anaesthesia resulted in more stable haemodynamic variables than spinal anaesthesia |
| Aoki and colleagues (1998)^2^ | Japan | Prospective study | 1. To evaluate the relation between the degree of highly echogenic material seen on TOE and the lipid-laden cells obtained from bronchoalveolar lavage 2. To assess the pathophysiology of fat embolism syndrome by comparing broncho-alveolar lavage fluid findings for fat embolism syndrome patients and those for patients who had undergo reamed intramedullary nailing | Femoral or tibial reamed intramedullary nailing | • Femoral fracture • Tibial fracture | Uncemented | • General | 15 | • Intraoperative TOE • Haemodynamic and pulmonary monitoring | • During reamed intramedullary nailing TOE showed highly echogenic material • Analysis of the debris retrieved from the right atrium showed fat • A significant increase in the percentage of fat cells was seen in patients with the most echogenic material seen on TOE |
| Barwood and colleagues (2000)^3^ | Australia | Retrospective study | 1. To determine the incidence and clinical characteristics of cardiorespiratory and vascular dysfunction in patients with femoral metastases, who underwent intramedullary nailing of the femur | Femoral reamed intramedullary nailing | • Femoral pathological (impending) fracture | Cemented + uncemented | • General • Spinal | 43 (45) | • Haemodynamic and pulmonary monitoring | • Systemic hypotension, desaturation or both (related to cement- or prosthesis insertion) were observed in 24.4% of the patients • The mortality in these episodes was high |
| Bhadani and colleagues (2024)^4^ | India | Prospective study | 1. To study the effect of bone cement on haemodynamics in hip arthroplasty in the eastern Indian population and compared it with the available literature | THA Hemiarthroplasty | • Femoral fracture • Malignant disease • Femoral pathological (impending) fracture • Osteoarthritis • Osteonecrosis | Cemented | • General | 72 | • Haemodynamic and pulmonary monitoring | • Incidence of BCIS grade 1 was 18.1% and grade 3 was 2.8%. BCIS grade 2 did not occur |
| Bisignani and colleagues (2008)^5^ | Italy | Prospective study | 1. To evaluate the relevance and the origin of embolization 2. To correlate the embolic events with hip arthroplasty 3. To establish the clinical impact of embolization | THA | • Hip fracture | Cemented + uncemented | • General | 40 | • Intraoperative TOE • Haemodynamic and pulmonary monitoring • Blood analysis | • Cemented arthroplasty was associated with more prolonged and more severe embolic events than when no cement is used • No relevant haemodynamic and pulmonary variations were observed • Analysis of the debris retrieved from the right atrium showed normal constituents of blood |
| Bökeler and colleagues (2022)^6^ | Germany | Retrospective study | 1. To investigate the influence of a comprehensive cementing technique on the development of BCIS in a geriatric patient population with hip fractures treated with cemented hemiarthroplasty | Hemiarthroplasty  • 3th generation cementing technology • 2nd generation cementing technology | • Femoral neck fracture | Cemented | • General | 92 | • Haemodynamic and pulmonary monitoring | • The overall incidence of BCIS was 58.1% and 34.7% during second generation cementing technique and third generation cementing technique, respectively |
| Bonfait and colleagues (2022)^7^ | France | Survey | 1. To assess the frequency of BCIS 2. To analyse risk factors related to the patient and to professional practices 3. To set out guidelines to reduce frequency and severity | THA • Primary scheduled • Acute (fractures) • Revision | • Femoral fracture • Other | Cemented + uncemented | Not reported | - | • Practices surveys with orthopaedic surgeons and anaesthesiologists | • BCIS was 6 times as frequent in traumatology as in scheduled surgery  • No cases of BCIS were reported in uncemented arthroplasty |
| Chen and colleagues (2016)^8^ | China | Retrospective study | 1. To evaluate the effect of the commonly used spinal-epidural anaesthesia and single epidural anaesthesia, on the incidence of BCIS in cemented hemiarthroplasty for hip fractures, using this severity classification of BCIS | Hemiarthroplasty | • Femoral neck fracture | Cemented | • Epidural • Spinal-epidural | 1210 | • Haemodynamic and pulmonary monitoring | • Overall incidence of BCIS was 72.2% • Single epidural anaesthesia was more associated with the development of the BCIS compared with spinal-epidural anaesthesia • Age, ASA classification and gender were observed to be risk factors |
| Christie and colleagues (1994)^9^ | United Kingdom | RCT | 1. To evaluate whether the insertion of cemented femoral protheses is specifically associated with more severe thromboembolism compared to uncemented femoral protheses | Hemiarthroplasty • Hastings (cemented) • Austin-Moore (uncemented) | • Femoral neck fracture | Cemented + uncemented | Not reported | 20 | • Intraoperative TOE • Haemodynamic and pulmonary monitoring • Blood analysis | • Cemented arthroplasty was associated with more prolonged and more severe embolic events than when no cement is used • Analysis of the debris retrieved from the right atrium during BCIS grade 2 showed fat |
| Christie and colleagues (1995) A^10^ | United Kingdom | RCT | 1. To describe the echocardiographic findings in operations which included medullary reaming | Femoral or tibial reamed intramedullary nailing Hemiarthroplasty | • Femoral pathological (impending) fracture • Femoral fracture • Tibial fracture | Cemented + uncemented | Not reported | 110 (111) | • Intraoperative TOE • Haemodynamic and pulmonary monitoring  • Blood analysis | • Embolic events were seen in 87.0% of the procedures • Cemented arthroplasty was associated with more prolonged and more severe embolic events than when no cement is used • Pulmonary responses were correlated with the severity of embolic phenomena • Analysis of the debris retrieved from the right atrium showed fat, bone marrow and aggregates of platelets |
| Christie and colleagues (1995) B^11^ | United Kingdom | RCT | 1. To establish whether preliminary lavage of the medullary canal during hemiarthroplasty reduced the embolic load and the severity of cardiorespiratory changes when cement is inserted | Hemiarthroplasty • Thorough lavage  • Minimal lavage | • Femoral neck fracture | Cemented | Not reported | 24 | • Intraoperative TOE • Haemodynamic and pulmonary monitoring | • Medullary lavage reduced the number of large emboli and the total duration of the embolic events • Medullary lavage led to less disturbance of pulmonary function • Analysis of the debris retrieved from the right atrium showed fat, bone marrow, air and fibrin |
| Chulsomlee  and colleagues (2022)^12^ | Thailand | Retrospective study | 1. To correlate the use of shaped-closed femoral stem and other perioperative risk factors with BCIS grade 2 or 3 2. To identify the prevalence of BCIS in the elderly patients | THA • Shape-closed femoral stem • Force-closed femoral stem | • Femoral neck fracture | Cemented | • General • Regional | 128 | • Haemodynamic and pulmonary monitoring | • The overall incidence of BCIS was 32.8% • The occurrence of BCIS grade 2 or 3 was associated with more perioperative complications • Age, use of shape-closed femoral stem design and preinjury Parker mobility score < 4 were observed to be risk factors |
| Clark and colleagues (2000)^13^ | United Kingdom | Prospective study | 1. To measure the effect of the introduction of methylmethacrylate during hemiarthroplasty of the hip on the CO | Hemiarthroplasty • Cemented  • Uncemented | • Femoral neck fracture | Cemented + uncemented | • General | 19 | • Haemodynamic and pulmonary monitoring | • Cementation led to a significant reduction in CO and SV • The HR and MAP were not affected |
| Clayer and colleagues (2007)^14^ | Sweden | Retrospective study | 1. To evaluate the incidence of desaturation hypotension or both, including significant cardiac events, in patients who are most at risk of such events: those undergoing osteosynthesis of lung cancer metastases in long bones | THA Hemiarthroplasty Intramedullary nailing | • Malignant disease | Cemented + uncemented | Not reported | 32 (34) | • Haemodynamic and pulmonary monitoring | • Systemic hypotension, desaturation or both (related to cement- or prosthesis insertion) were observed in 14.7% of the patients |
| De Froidmont and colleagues (2014)^15^ | Switzerland | Retrospective study | 1. To evaluate the role of anaphylaxis and pulmonary fat emboli in the pathophysiology of BCIS | • THA • Road-traffic accidents • Contrast medium administration • Laminectomy | • Femoral neck fracture | Cemented | Not reported | 50 | • Post-mortem examination | • Pulmonary embolism was determined to be the main cause of death in all 6 subjects who died intraoperatively |
| Dumanli Özcan and colleagues (2018)^16^ | Turkey | RCT | 1. To compare the effects of colloid preloading before or coloading simultaneously at cement implantation on BCIS development and haemodynamic parameters in patients who underwent total knee arthroplasty | Total knee arthroplasty with tourniquet  • Hydroxyethyl starch infusion 20 min before cementation • Hydroxyethyl starch infusion at time of cementation • Sodium chloride infusion during the anaesthesia management | Not reported | Cemented | • Spinal | 109 | • Haemodynamic and pulmonary monitoring | • The incidence of BCIS grade 1 was 20.6%, 14.7% and 70.7% in patients who received infusion 20 before cementation, at time of cementation or during anaesthesia management, respectively • Infusion with hydroxyethyl starch positively affected the haemodynamic variables compared to infusion with sodium chloride |
| Fernandez and colleagues (2025)^17^ | United Kingdom | Prospective study | 1. To observe differences in the timing and severity of haemodynamic instability between patients receiving cemented and uncemented hemiarthroplasty | Hemiarthroplasty | Not reported | Cemented + uncemented | • General • Spinal | 46 | • Haemodynamic and pulmonary monitoring | • Incidence of BCIS grade 1 was 15.4% and 5.0% during cemented and uncemented hemiarthroplasty, respectively. BCIS grade 2 or 3 did not occur  • Haemodynamic instability is more likely to occur within five minutes of cementing and at reduction of the hip • The fall in blood pressure were driven by a fall in CO and SV |
| Fujita and colleagues (2015)^18^ | Japan | Prospective study | 1. To monitor the blood pressure changes that occur after cementing during cemented THA involving the interface bioactive bone cement technique | THA (interface bioactive bone cement technique) | • Femoral neck fracture • Osteoarthritis • Rheumatoid arthritis • Osteonecrosis | Cemented | • General | 178 (204) | • Haemodynamic and pulmonary monitoring | • In most cases blood pressure increased after cementation |
| Funahashi and colleagues (2020)^19^ | Japan | Retrospective study | 1. To examine blood pressure around cement insertion during hemiarthroplasty in the elderly | Hemiarthroplasty | • Femoral neck fracture | Cemented | • Spinal | 430 | • Haemodynamic and pulmonary monitoring | • The maximum regulation ratio of patients with a >10% increase in blood pressure was significantly less than that of patients with a <10% change in blood pressure |
| Garcia-Mansilla and colleagues (2023)^20^ | Argentina | Retrospective study | 1. To investigate whether unfractionated heparin has a protective effect by decreasing the incidence of BCIS and other perioperative thromboembolic events | THA • Unfractionated heparin before cementation • No intraoperative unfractionated heparin | • Femoral neck fracture | Cemented | • General • Spinal | 273 | • Haemodynamic and pulmonary monitoring | • The overall incidence of BCIS was 18.7% • The use of unfractionated heparin was a risk factor |
| Hagio and colleagues (2003) A^21^ | Japan | Prospective study | 1. To examine the frequency and grade of and risk factors for embolic events during various intraoperative procedures involved in uncemented and cemented THA | THA | • Osteoarthritis  • Osteonecrosis • Rheumatoid arthritis | Cemented + Uncemented | • General-epidural | 88 | • Intraoperative TOE • Haemodynamic and pulmonary monitoring | • Cemented arthroplasty was associated with more prolonged and more severe echogenic events than when no cement is used • PaO_2_, arterial oxygen saturation and SBP significantly decreased during severe embolic events |
| Hagio and colleagues (2003) B^22^ | Japan | Prospective study | 1. To determine whether the incidence of preoperative pulmonary embolism during uncemented THA differed between the ROBODOC femoral milling system, and manual surgery | THA  • ROBODOC femoral milling system • Conventional arthroplasty | • Osteoarthritis | uncemented | • General-epidural | 71 | • Intraoperative TOE • Haemodynamic and pulmonary monitoring | • Conventional arthroplasty was associated with more severe embolic events than ROBODOC arthroplasty  • Severe embolic events led to a significant reduction in PaO_2_ and arterial oxygen saturation |
| Hall and colleagues (2017)^23^ | Canada | RCT | 1. To determine whether the use of the Reamer Irrigator Aspirator device resulted in a decreased amount of fat emboli compared with standard reaming | Femoral reamed intramedullary nailing • Reamer Irrigator Aspirator device • Standard reaming technique | • Femoral fracture | Uncemented | Not reported | 22 | • Intraoperative TOE • Haemodynamic and pulmonary monitoring • Blood analysis | • Reamer Irrigator Aspirator system led to a modest reduction of embolic debris during reaming and nailing  • The reduction of emboli in the Reamer Irrigator Aspirator group was not correlated with any difference in haemodynamic and pulmonary variables |
| Hananouchi  and colleagues (2006)^24^ | Japan | Prospective study | 1. To evaluate whether the suctioning technique reduced frequency of the severe embolic events on the TOE during insertion of the acetabular components without holes 2. To determine whether the suctioning technique prevented aggravation of the blood gas and blood pressure which are associated with the severe emboli | THA • Suctioning technique during cup insertion • Conventional cup insertion | • Osteoarthritis • Osteonecrosis | Cemented + uncemented | • General-epidural | 54 (60) | • Intraoperative TOE • Haemodynamic and pulmonary monitoring • Blood analysis | • Conventional arthroplasty was associated with more severe embolic events than when the suctioning technique was used • The suctioning technique prevented a decrease in PaO_2_ and blood pressure |
| Hård Af Segerstad and colleagues (2018)^25^ | Sweden | RCT | 1. To evaluate the differential effects of cemented versus uncemented total knee arthroplasty on the pulmonary haemodynamic response | Total knee arthroplasty • Cemented  • Uncemented | • Femoral neck fracture | Cemented + uncemented | • General | 22 | • Haemodynamic and pulmonary monitoring • Blood analysis | • The mean dose of norepinephrine significantly increased during the procedure in the cemented group  • PaO_2_ decreased significantly in the cemented group • The increase in MPAP was more pronounced in the cemented group |
| Hård Af Segerstad and colleagues (2019)^26^ | Sweden | RCT | 1. To evaluate the effects of inhaled prostacyclin versus inhaled saline on pulmonary vascular resistance index | Hemiarthroplasty • Intraoperative inhalation with prostacyclin  • Intraoperative inhalation with saline | • Femoral neck fracture | Cemented | • General | 22 | • Haemodynamic and pulmonary monitoring | • No significant differences in haemodynamic variables were seen between the prostacyclin group and saline group |
| Helttula and colleagues (2000)^27^ | Finland | Prospective study | 1. To analyse immediate preoperative, perioperative, and postoperative cardiac and pulmonary haemodynamic changes | Tibial reamed intramedullary nailing | • Tibial fracture | Uncemented | • General | 12 | • Haemodynamic and pulmonary monitoring | • During the nailing procedure, the MPAP was slightly increased |
| Helttula and colleagues (2006)^28^ | Finland | RCT | 1. To examine whether there are acute differences in cardiopulmonary variables between patients undergoing reamed or unreamed nailing 2. To identify the timepoints when the heart is most strained during the study | Femoral intramedullary nailing • Reamed  • Unreamed | • Femoral fracture | Uncemented | • General | 18 | • Haemodynamic and pulmonary monitoring | • No relevant haemodynamic and pulmonary variations were observed between the groups |
| Herrenbruck  and colleagues (2002)^29^ | United States | Retrospective study | 1. To evaluate whether patients with femoral metastatic disease and previously uninstrumented canals are at higher risk for adverse events | THA (long-stem) • Revision  • Primary | • Malignant disease • Other | Cemented | Not reported | 55 | • Haemodynamic and pulmonary monitoring | • Systemic hypotension, desaturation or both (related to cement- or prosthesis insertion) were observed in 38.2% of the patients • Risk factors for cement-associated adverse events are metastatic disease, femoral canals without previous instrumentation and longer stems |
| Jaffe and colleagues (2022)^30^ | United States | Retrospective study | 1. To describe the incidence of BCIS according to the classification defined by Donaldson *et al.* 2. To evaluate preexisting factors associated with BCIS as well as its impact on mortality | Hemiarthroplasty | • Femoral neck fracture | Cemented | • General • Regional | 69 | • Haemodynamic and pulmonary monitoring | • The overall incidence of BCIS was 35.0%, grade 2 and 3 was seen in 10.1% |
| Karanko and colleagues (2010)^31^ | Finland | Prospective study | 1. To survey pre- and postoperative haemodynamic and gas exchange in patients with cancer lungs and to compare them with previously published values in healthy patients with femoral shaft fractures 2. To examine if pathological haemodynamic and oxygenation trends surpassing the usual response to nailing can be found intraoperatively | Femoral reamed intramedullary nailing • Cancer patients • Healthy patients | • Femoral pathological (impending) fracture | Uncemented | • General | 20 | • Haemodynamic and pulmonary monitoring • Blood analysis | • During intramedullary nailing pulmonary shunting and the MPAP increased |
| Kato and colleagues (2002)^32^ | Japan | RCT | 1. To determine whether cardiopulmonary impairment during the period of tourniquet inflation was related to echogenic findings detected by TOE 2. To identify the composition of the echogenic material | Total knee arthroplasty  • With tourniquet • Without tourniquet | Not reported | Cemented + uncemented | • General | 46 | • Intraoperative TOE • Haemodynamic and pulmonary monitoring • Blood analysis | • TOE monitoring showed embolic material even while the tourniquet was inflated • Haemodynamic and pulmonary impairment was seen |
| Kaufmann and colleagues (2018)^33^ | Germany | RCT | 1. To evaluate whether optimising haemodynamic parameters by oesophageal Doppler monitor-guided goal-directed therapy before cement implantation would reduce the incidence of BCIS | THA  • Goal-directed therapy guided by oesophageal Doppler monitoring-based algorithm • Conventional fluid and haemodynamic management | • Femoral fracture • Osteoarthritis • Infectious • Osteonecrosis • Other | Cemented | • General-Regional | 90 | • Haemodynamic and pulmonary monitoring | • The overall incidence of BCIS in the conventional group was 46.7% and in the goal-directed therapy group 38.0%  • Goal-directed therapy guided by oesophageal Doppler monitoring led to better CO and SV-index at the time of cement insertion while MAP was comparable between groups |
| Kim and colleagues (2001)^34^ | South-Korea | Prospective study | 1. To determine if unilateral or bilateral simultaneous total knee arthroplasty procedures resulted in a differing incidence of fat embolization, degree of haemodynamic compromise, levels of hypoxemia, or mental status changes | Total knee arthroplasty with tourniquet (uni- or bilateral) • Cemented • Uncemented | • Osteoarthritis • Osteonecrosis • Rheumatoid arthritis | Cemented + uncemented | • Epidural | 200 (300) | • Haemodynamic and pulmonary monitoring • Blood analysis | • No relevant haemodynamic and pulmonary variations were observed between the groups  • Analysis of the debris retrieved from the right atrium showed fat |
| Kim and colleagues (2002)^35^ | South-Korea | RCT | 1. To determine if unilateral and bilateral simultaneous total knee arthroplasty resulted in different prevalences of fat embolization, different degrees of haemodynamic compromise, or different levels of hypoxemia or mental status changes 2. To investigate the prevalence of fat embolization in patients managed with cemented compared with uncemented stems | THA (uni- or bilateral) • Cemented • Uncemented | • Femoral neck fracture • Osteoarthritis • Osteonecrosis • Rheumatoid arthritis • Other | Cemented + uncemented | • Epidural | 156 (206) | • Haemodynamic and pulmonary monitoring • Blood analysis | • No significant difference was seen in the intraoperative haemodynamic values between the groups treated with unilateral and bilateral arthroplasty and between the groups treated with cemented and uncemented stems |
| Kim and colleagues (2008)^36^ | South-Korea | RCT | 1. To determine whether the prevalence of fat, bone-marrow-cell embolization or both would be lower in patients undergoing total knee arthroplasty with computer-assisted navigation than it would be in patients undergoing total knee arthroplasty with a conventional method of insertion of intramedullary alignment rods 2. To determine any risk factors for fat, bone-marrow-cell or both embolization | Total knee arthroplasty with tourniquet (uni- or bilateral) • Computer-assisted navigation  • Conventional method | • Osteoarthritis • Osteonecrosis • Rheumatoid arthritis | Cemented | • Epidural | 260 (420) | • Haemodynamic and pulmonary monitoring • Blood analysis | • During both computer-assisted navigation and conventional arthroplasty, the prevalence of fat embolization was increased compared with baseline • No relevant haemodynamic and pulmonary variations were observed  • Analysis of the debris retrieved from the right atrium showed fat |
| Koessler and colleagues (2001)^37^ | Germany | RCT | 1. To find correlations between the embolic events observed while using TOE and the cardiopulmonary function of the patients during the intraoperative and postoperative periods 2. To determine the efficacy of a modified surgical technique designed to limit the intramedullary pressure during insertion of the stem and to reduce intraoperative embolic events | THA • Modified cementing technique • Conventional cementing technique | • Osteoarthritis • Femoral fracture | Cemented | • General | 120 | • Intraoperative TOE • Haemodynamic and pulmonary monitoring | • The modified surgical technique resulted in less fat embolization  • A relationship between embolic events grade 2 and 3 and changes of haemodynamic and cardiorespiratory function were observed |
| Kotyra and colleagues (2010)^38^ | Sweden | Prospective study | 1. To evaluate the effects of cemented hemiarthroplasty on pulmonary haemodynamic, right ventricular performance, intrapulmonary shunting and physiological dead space | Hemiarthroplasty | • Femoral neck fracture | Cemented | • General | 15 | • Haemodynamic and pulmonary monitoring | • MAP decreased • The lung oxygenation index was impaired  • An increase was seen in physiological dead space and intra-pulmonary shunting |
| Lafont and colleagues (1997)^39^ | France | Prospective study | 1. To record evidence of embolism using TOE 2. To relate the embolic events seen on TOE to demographic data and the subsequent clinical course during THA | THA | Not reported | Cemented | • General | 48 | • Intraoperative TOE • Haemodynamic and pulmonary monitoring | • Echogenic material was observed during reaming and cementation of the acetabular and femoral components • No relationship was seen between embolic frequency or size and haemodynamic variables |
| Lamadé and colleagues (1995)^40^ | Germany | RCT | 1. To demonstrate a potential benefit of histamine-receptor-blocking agents | THA • Intravenous infusion with saline, clemastine and cimetidine  • Infusion with normal saline | • Femoral neck fracture • Osteoarthritis | Cemented | • General | 30 | • Haemodynamic and pulmonary monitoring • Blood analysis | • In both groups, a decrease in blood pressure was seen after insertion of the femoral prosthesis |
| Li and colleagues (2009)^41^ | China | Prospective study | 1. To observe the changes in MAP, HR and PaO2 during cemented THA 2. To evaluate the intraoperative safety of the third-generation cementing technique 3. To investigate whether the intraoperative risk is higher in acute femoral neck fracture patients than non-traumatic patients | THA • Third-generation cementing technique | • Femoral fracture • Osteoarthritis • Osteonecrosis • Rheumatoid arthritis | Cemented | • Spinal | 42 | • Haemodynamic and pulmonary monitoring • Blood analysis | • No relevant haemodynamic and pulmonary variations were observed |
| Li and colleagues (2020)^42^ | China | RCT | 1. To explore the effect of predictive nursing mode on preventing from BCIS in hemiarthroplasty | Hemiarthroplasty • Predictive nursing mode • Conventional treatment | Not reported | Cemented | • Spinal-epidural | 55 | • Haemodynamic and pulmonary monitoring | • SBP, HR and saturation were significantly were higher in the intervention group compared to the conventional group |
| López-Durán  and colleagues (1997)^43^ | Spain | Prospective study | 1. To detect haemodynamic changes and their consequences during THA | THA • Cemented  • Uncemented | • Osteoarthritis | Cemented + uncemented | • General | 30 | • Intraoperative TOE • Haemodynamic and pulmonary monitoring • Blood analysis | • No relevant haemodynamic and pulmonary variations were observed in cemented and uncemented THA • Echogenic material was seen on TOE during insertion of cement, implantation of the femoral component and reduction of the prosthesis |
| Lu and colleagues (2017)^44^ | China | Prospective study | 1. To detect the origin of the pulmonary embolus and identify relevant risk factors of pulmonary embolism in total knee arthroplasty | Total knee arthroplasty with tourniquet | • Osteoarthritis | Not reported | • General | 40 | • Intraoperative TOE • Haemodynamic and pulmonary monitoring • Blood analysis • Bone marrow analysis | • Embolic events were seen when the guiding apparatus was inserted, the prosthesis was being installed and seconds after tourniquet release • Total embolus quantity was positively correlated with age and the fat content of the bone marrow • MPAP was increased • Analysis of the debris retrieved from the right atrium showed fat |
| Malhotra and colleagues (2015)^45^ | India | RCT | 1. To evaluate whether total knee arthroplasty performed with computer-assisted surgery would result in a lower embolic burden compared with surgery with conventional instrumentation 2. To investigate the type of emboli generated during the procedure  3. To investigate the immediate clinical implications of the emboli | Total knee arthroplasty with tourniquet • Computer-assisted navigation  • Conventional method | Not reported | Cemented | Not reported | 57 | • Intraoperative TOE • Haemodynamic and pulmonary monitoring • Blood analysis | • Conventional total knee arthroplasty was associated with more severe and longer embolic events than computer-assisted surgery • No relevant haemodynamic variations were observed • Analysis of the debris retrieved from the right atrium showed fat and air |
| Miyamoto and colleagues (2017)^46^ | Japan | Prospective study | 1. To evaluate changes in intraoperative blood pressure and the incidence of major complications, including loss of consciousness, cardiac arrest, and arrhythmias | Hemiarthroplasty • Cemented  • Uncemented | • Femoral neck fracture | Cemented + uncemented | • General • Spinal | 164 | • Haemodynamic and pulmonary monitoring | • The overall incidence of BCIS was 29.0% and 21.8% in cemented and uncemented hemiarthroplasty, respectively  • In the cemented group, some patients experienced an increase in SBP |
| Motobe and colleagues (2004)^47^ | Japan | Prospective study | 1. To investigate potential effects of endogenous cannabinoids in patients undergoing cemented hip arthroplasty | THA or hemiarthroplasty • Cemented  • Uncemented | • Femoral neck fracture • Osteoarthritis • Rheumatoid arthritis | Cemented + uncemented | • General • General-epidural | 35 | • Haemodynamic and pulmonary monitoring • Blood analysis | • Overall incidence of BCIS was 100% in the cemented group and 0.0% in the uncemented group |
| Movrin  (2020)^48^ | Slovenia | RCT | 1. To compare the results of hemiarthroplasty using a cemented versus press-fit uncemented femoral stem while focusing on any differences in intraoperative events, functional outcomes, and rates of postoperative complications between groups | Hemiarthroplasty • Cemented • Uncemented | • Femoral neck fracture | Cemented | Not reported | 158 | • Haemodynamic and pulmonary monitoring | • Desaturation (related to cement- or prosthesis insertion) was observed in 10.1% and 0.0% of the patients in the cemented group and uncemented group, respectively  • Systemic hypotension (related to cement- or prosthesis insertion) was observed in 18.9% and 5.1% of the patients in the cemented group and uncemented group, respectively |
| Nolan  (1994)^49^ | United Kingdom | Prospective study | 1. To examine the effects of uncemented THA on PaO_2_ and MAP and to compare these with a cemented group | THA • Cemented  • Uncemented | Not reported | Cemented + uncemented | • General | 20 | • Haemodynamic and pulmonary monitoring • Blood analysis | • Cemented THA resulted in a decrease in PaO_2_ and an increase in MAP  • In uncemented THA PaO_2_ and MAP remained unchanged |
| Norris and colleagues (2001)^50^ | United States | Prospective study | 1. To investigate whether the amount of alveolar dead space changes with intramedullary nailing of femoral shaft fractures and during reaming 2. To explore whether other variables such as the Injury Severity Score, pulmonary injury, and pulmonary comorbidities may more accurately predict pulmonary dysfunction following intramedullary nailing of femoral shaft fractures | Femoral intramedullary nailing  • Extensive reaming • Minimal or no reaming | • Femoral fracture | Not reported | • General | 74 (80) | • Haemodynamic and pulmonary monitoring • Blood analysis | • Intramedullary nailing did not change the alveolar dead space significantly |
| Olsen and colleagues (2014)^51^ | Sweden | Retrospective study | 1. To estimate the incidence of BCIS in cemented hemiarthroplasty for hip fractures 2. To elucidate the risk factors for the development of this syndrome 3. To investigate the impact of BCIS for early (30 days) and late (1 year) mortality | Hemiarthroplasty | • Femoral neck fracture | Cemented | • General • General-regional | 1016 | • Haemodynamic and pulmonary monitoring | • The overall incidence of BCIS was 28.0% • Severe BCIS was associated with increased 30 day and one year mortality  • ASA classification was observed to be a risk factor |
| Olsen and colleagues (2020)^52^ | Sweden | Retrospective study | 1. To evaluate the role of bone cementation for the development of intraoperative haemodynamic and pulmonary derangement and its impact on postoperative mortality | Hemiarthroplasty | • Femoral neck fracture | Uncemented | • General • General-regional | 109 | • Haemodynamic and pulmonary monitoring | • Overall incidence of BCIS 17.0% • One year mortality was 15.0% |
| Park and colleagues (2015)^53^ | South-Korea | Prospective study | 1. To compare haemodynamic changes via the measurement of cardiac index and SV between the elderly (65–84 years) and the very elderly patients (≥ 85 years) | Hemiarthroplasty | • Femoral neck fracture | Cemented | • Spinal | 97 | • Haemodynamic and pulmonary monitoring | • SV and cardiac index were significantly lower • MAP and HR showed no significant differences |
| Parmet and colleagues (1994)^54^ | United States | Prospective study | 1. To determine the relationship between embolic patterns and alterations in pulmonary haemodynamic following tourniquet release 2. To determine embolic composition | Total knee arthroplasty with tourniquet | Not reported | Cemented | • General | 34 | • Intraoperative TOE • Haemodynamic and pulmonary monitoring • Blood analysis | • Analysis of the debris retrieved from the right atrium showed fresh thrombus • MAP increased after cementing  • SpO_2_ did not change |
| Parmet and colleagues (1995)^55^ | United States | Prospective study | 1. To determine the incidence of echogenic venous emboli in patients receiving extramedullary guided total knee arthroplasty | Total knee arthroplasty with tourniquet (extramedullary guided) | Not reported | Cemented | • General | 21 | • Intraoperative TOE • Haemodynamic and pulmonary monitoring | • TOE showed no echogenic material during surgery, only after tourniquet deflation • MAP and MPAP increased after cementing |
| Parmet and colleagues (1998)^56^ | United States | RCT | 1. To determine the incidence of large venous emboli in patients undergoing either intramedullary or extramedullary guided total knee arthroplasty without pneumatic tourniquet inflation | Total knee arthroplasty without tourniquet • Intramedullary alignment guide • Tibial extramedullary alignment guide | Not reported | Cemented | • General | 23 (24) | • Intraoperative TOE • Haemodynamic and pulmonary monitoring | • One third of knee replacements done without a tourniquet showed large emboli • Reducing marrow cavity invasion did not decrease the release of large emboli |
| Parvizi and colleagues (1999)^57^ | United kingdom | Retrospective study | 1. To determine the incidence of intraoperative mortality associated with all types of hip arthroplasty 2. To identify the risk factors for irreversible cardiovascular collapse | THA Hemiarthroplasty | • Hip fracture • Malignant disease • Other • Rheumatoid arthritis • Osteoarthritis | Cemented | • General • Regional | 29431 (38488) | • Haemodynamic and pulmonary monitoring | • The incidence of BCIS grade 3 in arthroplasty of any kind was 0.1% • No intraoperative deaths occurred in uncemented arthroplasties • Arthroplasty for a fracture was associated with increased mortality |
| Pitto and colleagues (1998)^58^ | Germany | RCT | 1. To assess the efficiency of the vacuum cementing technique developed to prevent the risk of intraoperative pulmonary embolism | THA • Vacuum cementing technique • Conventional cementing technique | • Osteoarthritis | Cemented | • General | 70 | • Intraoperative TOE • Haemodynamic and pulmonary monitoring • Blood analysis | • The vacuum cementing resulted in significantly fewer embolic events |
| Pitto and colleagues (1999) A^59^ | Germany | RCT | 1. To assess the relevance of drainage with vacuum suction placed along the linea aspera for the reduction of intramedullary pressure during the insertion of a cemented stem and for the prevention of the risk of embolism | THA  • Proximal drainage technique • Conventional cementing technique | • Osteoarthritis | Cemented | • General | 40 | • Intraoperative TOE • Haemodynamic and pulmonary monitoring • Blood analysis | • Conventional cementing technique was associated with more severe embolic events than when drainage of the proximal femur along the linea aspera was done • Pulmonary shunting increased significantly in the control group |
| Pitto and colleagues (1999) B^60^ | Germany | RCT | 1. To compare the effects of fixation of the femoral component without cement with those of fixation with a bone-vacuum cementing technique on the severity of embolic phenomena and cardiopulmonary impairment | THA • Uncemented procedure • Conventional cementing technique • Bone-vacuum cementing technique | • Osteoarthritis | Cemented + uncemented | • General | 60 | • Intraoperative TOE • Haemodynamic and pulmonary monitoring • Blood analysis | • Cemented arthroplasty was associated with more prolonged and more severe echogenic events than when no cement was used • Cardiopulmonary impairment was seen in the conventional cementing technique |
| Pitto and colleagues (2000)^61^ | Germany | RCT | 1. To verify the value of bone vacuum technique | THA • Bone-vacuum cementing technique • Conventional cementing technique | • Femoral neck fracture | Cemented | • General | 40 | • Intraoperative TOE • Haemodynamic and pulmonary monitoring • Blood analysis | • Conventional cementing technique was associated with more prolonged and more severe embolic events than when the vacuum cementing technique was used • Cardiopulmonary impairment was seen in the conventional cementing technique |
| Price and colleagues (2013)^62^ | United States | Retrospective study | 1. To identify intraoperative complications potentially attributable to the use of cemented long-stem femoral components 2. To identify early postoperative complications potentially attributable to the use of cemented long-stem femoral components | THA (long-stem) | • Femoral pathological (impending) fracture | Cemented | • General | 42 | • Haemodynamic and pulmonary monitoring | • Systemic hypotension, desaturation or both (related to cement- or prosthesis insertion) were observed in 18.2% of the patients |
| Qi and colleagues (2015)^63^ | China | Retrospective study | 1. To investigate the influence of bone cement implantation on haemodynamics 2. To investigate the preventive effect of epinephrine hydrochloride on pulmonary embolism | Hemiarthroplasty with flush of the medullary cavity with  • Epinephrine hydrochloride  • Normal saline | • Femoral neck fracture | Cemented | • General | 128 | • Haemodynamic and pulmonary monitoring | • Flushing the medullary cavity with normal saline led to a decrease of blood pressure and SpO_2_ • Flushing the medullary cavity with epinephrine hydrochloride did not let to any significant changes in haemodynamic variables |
| Randall and colleagues (2006)^64^ | United States | Retrospective study | 1. To evaluate whether aggressive medullary lavage, intraoperative long-tip canal suction and low-viscosity methylmethacrylate, could minimise the morbidity of patients with a cemented long-stem arthroplasty | THA (long-stem) | • Femoral pathological (impending) fracture | Cemented | Not reported | 27 (29) | • Haemodynamic and pulmonary monitoring | • Systemic hypotension (related to cement- or prosthesis insertion) was observed in 13.8% of the patients  • No desaturation (related to cement- or prosthesis insertion) was observed |
| Rao and colleagues (2022)^65^ | United States | Retrospective study | 1. To identify risk factors and potential mitigating factors of BCIS | Hip arthroplasty Knee arthroplasty | • Malignant disease | Cemented | • General • General-regional • General-epidural | 67 | • Haemodynamic and pulmonary monitoring | • The overall incidence of BCIS was 46.2%  • General anaesthesia was a risk factor for the development of BCIS |
| Rassir and colleagues (2021)^66^ | The Netherlands | Retrospective study | 1. To report the incidence of BCIS 2. To determine whether severe BCIS is associated with an increased risk of death within 30 days of surgery 3. To identify factors associated with the development of severe BCIS | Hip, knee and shoulder arthroplasty | Not reported | Cemented | • General • Spinal | 3010 (3294) | • Haemodynamic and pulmonary monitoring | • The overall incidence of BCIS in cemented hemiarthroplasty, total hip and total knee arthroplasty were 30.8%, 24.4% and 27.5%, respectively • Severe BCIS was associated with an increased risk of death within 30 days of surgery • Age, ASA classification and renal impairment were observed to be risk factors |
| Ries and colleagues (1998)^67^ | United States | RCT | 1. To determine the effects of a fluted compared with a round femoral intramedullary alignment rod on intramedullary pressure and pulmonary shunting | Total knee arthroplasty with tourniquet (unilateral or simultaneous bilateral) • Fluted intramedullary alignment rod • Round intramedullary alignment rod | Not reported | Cemented | • Spinal | 48 (68) | • Pulmonary monitoring • Blood analysis | • A trend toward a lower change in intraoperative shunt was observed in unilateral surgeries done with the fluted rod |
| Robinson and colleagues (2001)^68^ | United Kingdom | Prospective study | 1. To quantify the coagulative and cardiorespiratory response to nailing of isolated fractures, using sensitive, invasive monitoring 2. To examine the correlation of the cardiorespiratory responses with the extent of the embolic load produced by nailing 3. To investigated whether there was an association between the changes in cardiorespiratory parameters and the development of post operative respiratory compromise | Intramedullary nailing for  • Isolated traumatic tibial fracture • Isolated traumatic femoral fracture • Femoral pathological (impending) fracture | • Femoral or tibial pathological (impending) fracture | Uncemented | • General | 84 | • Intraoperative TOE • Haemodynamic and pulmonary monitoring • Blood analysis | • A significant increase was seen in the amount, size and duration of embolic events during insertion of the guide-wire, reaming and insertion of the nail, this was more pronounced in patients with pathological fractures of the femur • During reaming there was a positive correlation between the severity of the embolic response and the increase in MPAP • In patients with a pathological fracture of the femur nailing produced hypoxic episodes |
| Rutter and colleagues (2014)^69^ | United Kingdom | Surveillance study | 1. To estimate the risk of death or severe harm due to BCIS | Hemiarthroplasty | • Femoral neck fracture | Cemented | Not reported | 62 | • Review of the National Reporting and Learning System | • Death or severe harm associated with the use of cement occurred in every 2900 surgeries |
| Santos and colleagues (2018)^70^ | Brazil | Retrospective study | 1. To propose a resuscitation algorithm for the event of cardiogenic shock during or shortly after prosthesis insertion 2. To investigate whether the use of ECMO as a complementary therapy to mechanical ventilation would improve the outcomes of patients presenting BCIS reducing the risk of mortality | THA  Hemiarthroplasty | Not reported | Cemented + uncemented | Not reported | 658 | • Haemodynamic and pulmonary monitoring • Multidisciplinary discussion | • Patients that do not respond to standard management procedures and persist with hypoxemia may improve their survival with temporary ECMO support |
| Schwarzkopf  and colleagues (2019)^71^ | United States | Retrospective study | 1. What is the occurrence of BCIS in cancer patients after hip arthroplasty? 2. What are the risk factors in cancer patients for the development of this syndrome? 3. What is the outcome for cancer patients with BCIS? | THA Hemiarthroplasty | • Femoral pathological (impending) fracture | Cemented | • General | 374 | • Haemodynamic and pulmonary monitoring | • The overall incidence of BCIS was 74.3%  • Age was observed to be a risk factor |
| Singh and colleagues (2018)^72^ | Malaysia | RCT | 1. To evaluate the effectiveness of distal femoral canal decompression in reducing the risk of cardiopulmonary events 2. To determine the quantum of emboli produced 3. To determine the procedure during the surgery during which the emboli are produced | THA (long-stem) • Decompression technique • Conventional technique | • Malignant disease | Cemented | • General | 32 | • Intraoperative TOE • Haemodynamic and pulmonary monitoring | • A decrease in blood pressure >40 mmHg was seen in 31.3% and 75.0% during the vacuum technique and conventional technique, respectively • Cementation, insertion of the stem and relocating the hip resulted in the highest amount embolic events |
| Soleimanha  and colleagues (2014)^73^ | Iran | Prospective study | 1. To assess the cardiovascular index changes during cemented hip hemiarthroplasty | Hemiarthroplasty | • Femoral neck fracture | Cemented | • General | 72 | • Haemodynamic and pulmonary monitoring | • MAP and SBP decreased immediately after cementation • Twelve patients showed arrhythmia during cementation |
| Song and colleagues (2014)^74^ | South-Korea | RCT | 1. To identify the cardiovascular and respiratory effects of pressurised cement insertion | Hemiarthroplasty • Pressurisation technique • Non-pressurisation technique | Not reported | Cemented | • Spinal | 24 | • Haemodynamic and pulmonary monitoring | • SBP and MAP decreased after cementation in both groups |
| Takashina and colleagues (2007)^75^ | Japan | Prospective study | 1. To determine the incidence of bone marrow embolism associated with acetabular prosthesis insertion 2. To evaluate the effects of intramedullary decompression of the acetabulum in suppressing bone marrow embolism | Part 1 THA • Two-piece type prosthesis with one hole • Two-piece type prosthesis with holes • One-piece type prosthesis without holes  Part 2 THA (one-piece type acetabular prosthesis without holes) • Intramedullary decompression • Press-fit method | Not reported | Uncemented | • General-epidural | 150 + 60 | • Intraoperative TOE • Haemodynamic and pulmonary monitoring | • One-piece type prosthesis without holes was associated with more severe embolic events compared with two-piece prosthesis with holes • Conventional cementing technique was associated with more severe embolic events than when the decompression technique was used |
| Udogwu and colleagues (2025)^76^ | United States | Survey | 1. To assess the level of experience and amount of concern among musculoskeletal tumour surgeons regarding BCIS 2. To evaluate the biomechanical properties of a novel stem design compared to a standard stem design 3. To measure the intramedullary insertion pressures during cementation of a novel stem design compared to a standard stem design | Not reported | Not reported | Cemented | Not reported | - | • Practices surveys with Musculoskeletal Tumour Society (MSTS) surgeons | • Sixty-three (58.9%) respondents had experienced BCIS grade 3 during their practice |
| Ukaj and colleagues (2021)^77^ | Slovenia | Prospective study | 1. Do change in the intramedullary pressure after implantation of a cemented femoral stem has a different impact on hypoxia in elderly and young patients? | THA | Any diagnosis other than • Periprosthetic fracture • Femoral pathological (impending) fracture  • Revision | Cemented | • Spinal | 25 | • Haemodynamic and pulmonary monitoring | • The overall incidence of BCIS was 23.1% and 100% in patients younger and older than 66.5 years, respectively |
| Urban and colleagues (1996)^78^ | United States | Prospective study | To assess the right ventricular function using a right ventricular ejection fraction pulmonary artery catheter and TOE | THA | Not reported | Cemented | • General-epidural • Epidural | 18 | • Intraoperative TOE • Haemodynamic and pulmonary monitoring | • All patients showed echogenic material on TOE  • All patients showed haemodynamic changes, but most of these were small and clinically insignificant |
| Wang and colleagues (2022)^79^ | China | RCT | 1. To investigate the therapeutic effect of phenylephrine combined with goal-directed fluid therapy in elderly patients undergoing total knee arthroplasty | THA with goal-directed fluid therapy  • With phenylephrine  • Without phenylephrine | Not reported | Cemented | • General | 80 | • Haemodynamic and pulmonary monitoring | • Intraoperative HR and MAP in the experimental group were more stable than in the control group |
| Weingärtner  and colleagues  (2021)^80^ | Germany | Retrospective study | 1. To investigate the influence of BCIS on the clinical course in patients with a proximal femur fracture and with implantation of a cemented hemiarthroplasty 2. To determine the risk factors for the occurrence of BCIS | Hemiarthroplasty | • Femoral neck fracture | Cemented | • General | 208 | • Haemodynamic and pulmonary monitoring | • The incidence of overall BCIS was 37.0%  • BCIS was associated with an increased in-hospital mortality rate • Age and ASA classification were observed to be risk factors |
| Yang and colleagues (2021)^81^ | Taiwan | Retrospective study | 1. To investigate the general incidence of BCIS in bone tumour surgeries 2. To identify the severity and the clinical course of BCIS according the preexisting classification 3. To clarify whether there is any risk factor of BCIS for bone tumour surgeries | • Cementation alone • Cementation with plate and screws  • Cementation with intramedullary nailing • Cementation with arthroplasty | • Malignant disease • Femoral pathological (impending) fracture | Cemented | Not reported | 88 | • Haemodynamic and pulmonary monitoring | • The overall incidence of BCIS was 26.1%  • Risk factors for the development of BCIS were lung cancer or lung metastasis |
| Yektaş and colleagues (2015)^82^ | Turkey | RCT | 1. To assess the effect of pheniramine maleate and dexamethasone on incidence of hypotension, bradycardia and hypercarbia associated with BCIS | Partial hip prosthesis • Intravenous infusion with Pheniramine maleate and dexamethasone mixture  • Intravenous infusion with normal saline | • Femoral neck fracture | Cemented | • Spinal | 40 | • Haemodynamic and pulmonary monitoring • Blood analysis | • The dose of adrenaline and atropine used after cementation was significantly increased in the saline group • A significant difference between SpO_2_ values before and after cementation were seen in the saline group, which was not seen in the intervention group |
| Yuenyongviwat and colleagues (2024)^83^ | Thailand | Retrospective study | To assess whether the BCIS incidence in patients with preexisting heart disease undergoing cemented bipolar hemiarthroplasty is higher than that of patients without preexisting heart disease | Hemiarthroplasty • Preexisting heart disease • No preexisting heart disease | Not reported | Cemented | Not reported | 311 | • Haemodynamic and pulmonary monitoring | • Incidence of BCIS grade 1 was 4.2%. BCIS grade 2 and 3 did not occur • Preexisting heart disease was not a risk factor for the development of BCIS |
| Zastrow and colleagues (2025)^84^ | United States | Retrospective study | 1. To examine the relationship between anaesthetic regimen and the incidence of BCIS in patients undergoing cemented hemiarthroplasty for hip fractures | Hemiarthroplasty • General • General-regional • Spinal | • Femoral neck fracture | Cemented | • General • General-regional • Spinal | 137 | • Haemodynamic and pulmonary monitoring | • Incidence of BCIS grade 1 was 35.0%, grade 2 was 6.6% and grade 3 was 0.7% • Compared with neuraxial anaesthesia, general anaesthesia and general plus regional anaesthesia were associated with 6.8 (95% CI, 1.83 to 25.57) and 5.5 (95% CI, 1.57 to 18.99) times greater odds of BCIS, respectively |
| Zhao and colleagues (2015)^85^ | China | RCT | 1. To evaluate the effect of medullary cavity irrigation on fat emboli during total knee arthroplasty | Total knee arthroplasty with (in some procedures) tourniquet • Medullary canal saline irrigation • Without irrigation | • Osteoarthritis | Cemented | Not reported | 30 | • Intraoperative TOE • Haemodynamic and pulmonary monitoring | • Medullary irrigation could significantly reduce the formation of fat emboli • No relevant haemodynamic and pulmonary variations were observed between the groups |
